# Elevated Serum 25-Hydroxyvitamin D Levels Are Associated with Improved Bone Formation and Micro-Structural Measures in Elderly Hip Fracture Patients

**DOI:** 10.3390/jcm8111988

**Published:** 2019-11-15

**Authors:** Deepti K. Sharma, Rebecca K. Sawyer, Thomas S. Robertson, Roumen Stamenkov, Lucian B. Solomon, Gerald J. Atkins, Peter M. Clifton, Howard A. Morris, Paul H. Anderson

**Affiliations:** 1 Health and Biomedical Innovation, School of Pharmacy and Medical Sciences, University of South Australia, Adelaide 5001, Australiarebecca.sawyer@unisa.edu.au (R.K.S.); gerald.atkins@adelaide.edu.au (G.J.A.); peter.clifton@unisa.edu.au (P.M.C.); 2 Royal Adelaide Hospital, Adelaide 5001, Australiaroumen.stamenkov@sa.gov.au (R.S.); bogdan.solomon@sa.gov.au (L.B.S.); 3 Centre for Orthopaedic and Trauma Research, The University of Adelaide, Adelaide 5001, Australia

**Keywords:** 25-hydroxyvitamin D, bone surface/bone volume ratio, plate-like trabecular structure, mean wall thickness, CYP27B1, CYP24A1

## Abstract

Vitamin D, along with calcium, is generally considered necessary for bone health and reduction of fractures. However, he effects of improving vitamin D status have not always been observed to improve bone mineral density (BMD). We have investigated whether varying vitamin D status in humans, as measured by serum 25(OH)D levels, relate to micro-structural and histomorphetric measures of bone quality and quantity, rather than density. Intertrochanteric trabecular bone biopsies and serum samples were collected from patients undergoing hip arthroplasty (65 females, 38 males, mean age 84.8 ± 8.3 years) at Royal Adelaide Hospital. Estimated GFR, serum ionized calcium, alkaline phosphatase, albumin, supplement and medication intake prior to surgery were taken from patient case records. Serum 25(OH)D, 1,25(OH)2D, and parathyroid hormone (PTH) levels were measured by immunoassays. Trabecular bone structural indices were determined by high-resolution micro-CT. Mean wall thickness (MWT) was measured on toluidine blue-stained histological sections. Bone mRNA levels for vitamin D metabolising enzymes CYP27B1 and CYP24A1 were measured by qRT-PCR. While serum 25(OH)D levels did not associate with bone volume/tissue volume (BV/TV%), serum 25(OH)D levels were strongly and independently associated with MWT (*r* = 0.81 *p* < 0.0001) with values significantly greater in patients with higher serum 25(OH)D levels. Furthermore, serum 25(OH)D levels were negatively associated with Bone Surface/Bone Volume (BS/BV) (*r* = −0.206, *p* < 0.05) and together with bone CYP27B1 and CYP24A1 mRNA accounted for 10% of the variability of BS/BV (*p* = 0.001). These data demonstrate that serum 25(OH)D is an independent positive predictor of micro-structural and bone formation measures and may be dependent, in part, on its metabolism within the bone.

## 1. Introduction

Vitamin D insufficiency and inadequate dietary calcium intake are prevalent worldwide and considered to be a public health issue. Inadequate vitamin D levels have been associated with poor muscle function, increased risk of falls, and osteoporotic fractures. Randomized controlled trials of vitamin D and calcium supplementation show divergent results depending on the clinical outcomes investigated. Studies which have evaluated bone mineral density as an end-point show minimal effect of supplementation [1] whereas studies which have investigated fracture as the clinical outcome show significant reductions in risk of fractures only when vitamin D and calcium are supplemented together but not when vitamin D is supplemented alone [2]. A statistically significant reduction of 30% in the risk of hip fracture and a 15% reduction in the risk of total fractures were reported in a recent meta-analysis of randomized controlled trials on calcium and vitamin D supplementation, which included 30,970 participants [2]. In spite of the available positive evidence, the significance of vitamin D in the prevention and treatment of osteoporosis with or without calcium supplementation remains debated, and its role in maintenance of bone health is yet to be fully explicated [3].

All current clinical evidence indicates that the beneficial effects of vitamin D on bone are related to the serum levels of the prohormone 25-dihydroxyvitamin D_3_ (25(OH)D) and not 1,25-dihydroxyvitamin D_3_ (1,25(OH)_2_D), the active hormone, an apparent conundrum for the endocrine paradigm of vitamin D activity. In fact, the administration of 1,25(OH)_2_D has been shown to be detrimental for bone health [4]. Direct effects of 25(OH)D in bone have been demonstrated with the three major bone cell types, i.e., osteoblasts, osteocytes and osteoclasts expressing genes for vitamin D metabolizing enzymes, 25-hydroxyvitamin D 1∝-hydroxylase (CYP27B1), the enzyme that catalyzes the conversion of 25(OH)D to 1,25(OH)_2_D and 25-hydroxyvitamin D 24-hydroxylase (CYP24A1), the enzyme that catalyzes the degradation of 1,25(OH)_2_D as well as vitamin D receptor (VDR) [5,6]. The demonstration that 25(OH)D is metabolized within osteoblasts and that this is required for mineralization, suggests that 25(OH)D could promote within bone mineralization in humans, independent of circulating 1,25(OH)_2_D levels, which provides a physiological context for this clinical observation, i.e., beneficial effect of prohormone 25(OH)D [5,6].

The aim of the present study was to investigate the relationships between serum 1,25(OH)_2_D, 25(OH)D, parathyroid hormone (PTH), bone turnover markers on femoral bone structure in a cross-sectional study of elderly hip fracture patients. We also investigated the contribution of the bone CYP27B1 and CYP24A1 mRNA levels on the role of 25(OH)D in determining bone structure variables. We hypothesize that adequate vitamin D status results in local metabolism of 25(OH)D to 1,25(OH)_2_D within bone, resulting in measures of improved bone health.

## 2. Experimental Section

### 2.1. Bone and Serum Specimens

Intertrochanteric trabecular bone biopsies, serum and presurgical radiographs were collected from 103 patients (65 females, 38 males, age 85.04 ± 8.4) undergoing hip arthroplasty at Royal Adelaide Hospital (Adelaide, South Australia) between August 2015 and January 2017 due to experiencing a fracture of the neck of femur (NOF). During preoperative preparation, patients or next of kin were asked to participate in this study, and written consent was obtained. Date of surgery, patient age, weight, height, comorbidities, eGFR (MRD-EPI equation), medication history, total plasma levels of ionized calcium (estimated) [7], albumin and total alkaline phosphatase were collected from patient case reports. Patients that were being treated with anti-resorptive or calcitriol therapy were excluded from the study. The ethics approval for this study was obtained from Royal Adelaide Hospital Ethics Committee (Protocol no 130114; HREC/13/RAH/33), and institutional ethics approval was obtained from University of South Australia human ethics committee (HREC 35360).

### 2.2. Serum Analyses

Single blood specimen was collected at the time of surgery in SST tubes (serum separator tubes), centrifuged at 5000 rpm for 10 min to collect serum and stored at −80 °C until required. Serum samples were analyzed by chemiluminescent immunoassay (Diasorin Liaison) for 25(OH)D, 1,25(OH)_2_D, intact PTH, osteocalcin and bone-specific alkaline phosphatase.

### 2.3. Bone Biopsy Prepareation

The trabecular bone biopsies were cut into two halves with one half fixed in formalin (10% formalin) for 7 days at 4 °C followed by storage in 70% ethanol at room temperature until microstructural analyses were conducted. The other half was submerged in RNA later and stored at −80 °C for gene expression analyses.

### 2.4. Bone Biopsy Micro-Computed Tomography

Trabecular bone specimens were assessed for bone mass and bone quality variables with the 1076 Bruker micro-CT imaging system. The X-ray tube was operated at 60 KV and 167 µA with a resolution of 18 µm with a 0.5 mm aluminum filter and exposure time of 1770 ms. The images were reconstructed using the CTAn software (v1.13, Kontich, Belgium) after correcting for smoothing (1), beam hardening (30%) and ring artifact reduction (20). The specimens varied considerable in volume (1093.7 ± 511.2 mm^3^; range: 291.7–1078.8 mm^3^); hence, using a uniformly sized region of interest could have potentially oversampled the shorter bone and undersampled the longer bone. Hence, a slice-by-slice analysis tracing approach was adopted to maximize the volume available for analysis which included all trabecular bone and excluded cortical bone and marrow space. Morphometric indices were determined using an adaptive thresholding algorithm with a pre-thresholding of 60 to 255 and a radius of 8. Variables such as bone volume/tissue volume (BV/TV%), trabecular number (Tb.N), and trabecular separation (Tb.Sp) describe trabecular bone quantity or mass. BV/TV% and Tb.N are measures of the amount of trabecular bone within a selected volume of cancellous tissue. However, in addition to the quantity of bone, the trabecular bone quality plays a critical role in determining bone strength and is determined by the three-dimensional orientation and connectivity of individual trabeculae. Trabecular bone quality measures include bone surface/bone volume ratio (BS/BV) and trabecular thickness (Tb.Th). The measurement of BS/BV provides an integrated measure of thickness and texture of the bone.

### 2.5. Bone Biopsy Histology

**Figure A1:**
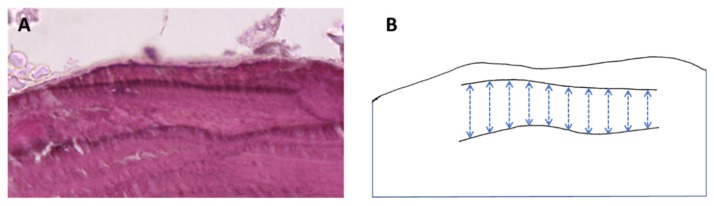
(**A**) Representative histological section indicating packet of trabecular bone and (**B**) representative measurements between cement lines.

Trabecular bone biopsies were then processed through 70% to 100% ethanol to dehydrate the sample and then placed in 5 mL solution containing 100% methylmethacrylate (MMA) and 10% *v*/*v* polyethylene glycol (PEG) and stored at room temperature for 10–14 days. Resin embedding then occurred by a solution of 100% MMA, 10% PEG and 0.4% peroxydicarbonate. Biopsies were submerged in 5 mL of this solution and placed in at 37 °C for 24 h to allow the resin to harden. A volume of 5 µm thick decalcified sections were cut and stained by Haematoxylin and Eosin [8]. Mean wall thickness, a measure of completed packets of bone remodeling, were measured in each section (Figure A1) (adapted from [9]). Wall thickness was defined as a complete trabecular bone packet on straight, rod-like trabecular structures. Five sites (10 measurements per site) per section were obtained between perpendicular cement lines beneath bone where no resorption of osteoid formation occurred.

### 2.6. Rodiographic Cortical Bone Analyses

**Figure A2:**
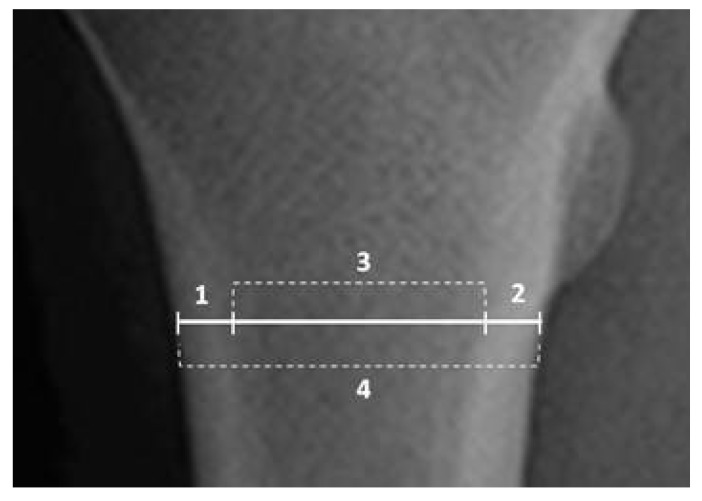
Representative femoral Anterior–Posterior plain radiograph indicating the measures of lateral cortical thickness (1), medial cortical thickness (2), M-L medullary width (3) and femoral width (4).

Cortical bone measurements: Anterior-Posterior plain radiographs of the contralateral uninjured limb were used to measure average medial (M) and lateral (L) cortical thicknesses and M-L medullary width and femoral width using CARESTREAM software (Figure A2).

### 2.7. Bone mRNA Analyses

Total RNA was extracted from the pulverized bone using the TRIZOL reagent (Life-technologies, Carlsbad, CA, USA), and cDNA samples were synthesized (Superscript-III kit, Life Technologies). The mRNA expression of genes of interest was measured by real-time PCR using iQ SYBR Green Supermix (Bio-Rad Laboratories, Hercules, CA, USA), as per the manufacturer’s instructions. Relative gene expression was quantified delta-CT method and normalizing to the expression of β-actin mRNA. The primer sequence for CYP27B1 is TGGCCCAGATCCTAACACATTT (forward) GTCCGGGTCTTGGGTCTAACT (reverse) CYP24A1 CCTGCTGCCAGATTCTCTGGAA (forward) TTGCCATACTTCTTGTGGTACTCC (reverse) and Beta actin CTGCCCTGAGGCACTCTT (forward) AGTTTCGTGGATGCCACAGG (reverse).

### 2.8. Statistical Analyses

Statistical analysis was performed using the SPSS statistical Package (IBM SPSS Stats 25, St. Leonards, Australia). Some of the variables were not normally distributed, hence values are expressed as median and interquartile range. Spearman’s correlation coefficient was calculated to determine correlations between structural, metabolic and gene expression variables. The metabolic variables which correlated with structural variables were tested through step-wise multiple linear regression analyses to identify metabolic determinants of bone mass and bone quality variables. Independent samples Kruskal–Wallis test was used to compare bone structural and quality variables between various 25(OH)D intervals. The significance level was set at 0.05.

## 3. Results

### 3.1. Biochemical Analyses

The median serum 25(OH)D levels were 52.7nmol/ L (Table 1). Serum 25(OH)D levels correlated positively with circulating 1,25(OH)_2_D (*r* = +0.311, *p* < 0.05) suggesting substrate insufficiency in a significant proportion of the patients. Serum 25(OH)D levels were not associated with eGFR or other measured biochemical parameters. However, serum 1,25(OH)_2_D levels were determined by a combination of serum 25(OH)D, PTH levels and eGFR (Multiple *R*^2^ = 0.235, *p* < 0.01) (Table 2). Negative correlations were observed between eGFR and age (*r* = −0.464, *p* < 0.001) and eGFR and PTH (*r* = −0.387, *p* < 0.001), consistent with a decline in renal function with age resulting in higher PTH values, which contributed to increased serum 1,25(OH)_2_D levels. Serum osteocalcin, a marker of bone turnover, correlated negatively with eGFR (*r* = −0.369, *p* < 0.001) suggesting that compromised kidney functions in these patients is associated with higher bone turnover.

### 3.2. Relationship between Serum Vitamin D Levels and Bone Structure and Histomorphometric Measures

**Figure 1:**
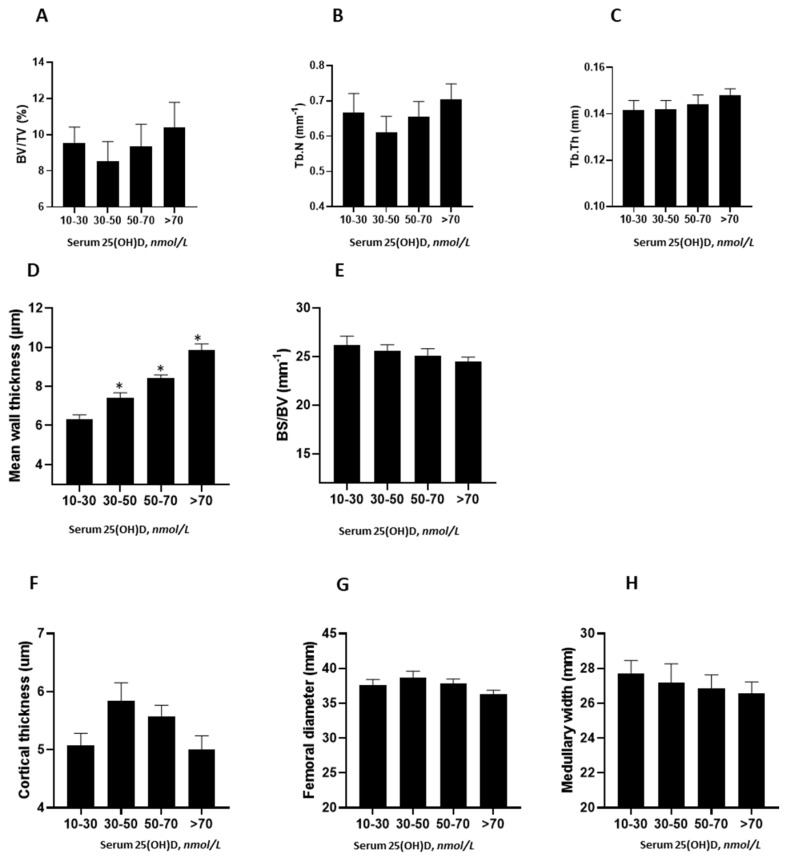
Trabecular and cortical bone structural variables across 25-dihydroxyvitamin D3 (25(OH)D intervals with (**A**) BV/TV (bone volume/tissue volume), (**B**) Tb.N (trabecular number) and (**C**) Tb.th (trabecular thickness), (**D**) MWT (mean wall thickness), (**E**) BS/BV (bone surface/bone volume), (**F**) cortical thickness, (**G**) femoral diameter and (**H**) medullary width. Mean +/− SEM; *n* = 103; * *p* < 0.05 vs. 10–30 nmol/L serum 25(OH)D interval.

**Figure A3:**
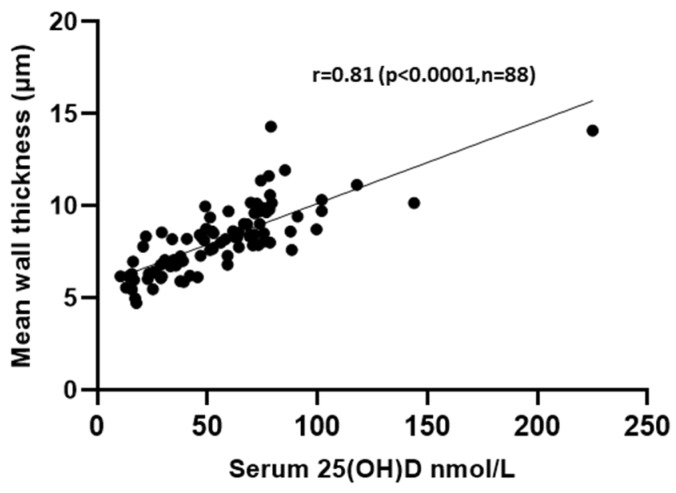
Scatter plot between serum 25(OH)D and mean wall thickness.

Serum 25(OH)D levels did not correlate with the bone structure variables of BV/TV%, Tb.Th and Tb.N by either linear analyses nor when serum 25(OH)D levels were grouped by intervals of 25(OH)D adequacy ranging from deplete to replete (Figure 1A–C). However, a trend for serum 25(OH)D levels association with Tb.Th was observed (*r* = 0.19, *p* = 0.06). Neither serum 1,25(OH)_2_D or PTH correlated with BV/TV%, Tb.N or Tb.Th. Mean wall thickness (MWT), a dynamic marker of completed bone formation, increased consistently and significantly with increasing vitamin D levels (Figure 1D, Table A1). MWT trended to be higher in patients with 25(OH)D greater than 70 nmol/L as compared to patients with serum 25(OH)D between 30–50 nmol/L (*p* = 0.021, adjusted *p* = 0.12) (Table A1). Furthermore, MWT exhibited a positive linear correlation with 25(OH)D levels (*r* = 0.810, *p* < 0.0001) (Figure A3). Although, MWT was also correlated with serum 1,25(OH)_2_D levels, only serum 25(OH)D determined MWT values in a multiple linear regression model (Table 3). Bone surface/bone volume (BS/BV) ratio, a measure of bone texture and a surrogate marker of plate-like trabecular structure, was negatively associated with serum 25(OH)D (*r* = −0.206, *p* < 0.05), indicative of higher vitamin D levels resulting in more plate-like structures. However, the BS/BV values did not reach statistical significance when divided by serum 25(OH)D intervals (Figure 1E). Neither serum 1,25(OH)_2_D nor PTH correlated with BS/BV (*p* = 0.9 and 0.8, respectively). With respect to cortical bone measurements, serum 25(OH)D levels correlated negatively with total femoral diameter (*r* = −0.239, *p* = 0.018) and trend only for medullary width (*r* = −0.169, *p* = 0.09). None of the cortical parameters differ significantly across vitamin D intervals (Figure 1F,H). Neither serum 1,25(OH)_2_D nor PTH contributed to the variation in any cortical measure.

### 3.3. Contribution of CYP27B1 and CYP24A1 mRNA Expression in Bone to Histomorphometric Measures

To determine whether bone-specific expression of vitamin D metabolizing enzymes, CYP27B1 and CYP24A1 contribute to the relationship of serum 25(OH)D with either MWT or BS/BV, multiple linear regression analyses were carried out to investigate the variance accounted by these measures. Bone CYP27B1 and CYP24A1 mRNA levels did not contribute to the serum 25(OH)D determination of MWT (Table 4). However, bone CYP24A1 mRNA contributed positively (*p* = 0.02) and CYP27B1 mRNA negatively; as a trend, only (*p* = 0.1) contributed to the serum 25(OH)D determination of BS/BV (Table 5).

## 4. Discussion

Vitamin D deficiency, as measured by serum 25(OH)D levels, has been shown to be associated with increased risk of hip fracture in the elderly [10]. Despite this knowledge, questions remain as to mechanisms by which higher 25(OH)D levels improve skeletal health [11], especially since levels above 20 nmol/L are considered adequate for renal 1,25(OH)_2_D synthesis and for intestinal calcium absorption [12]. In clinical studies, the effects of vitamin D status and nutritional supplementation in skeletal health have been largely limited to measures of BMD and fracture incidence. However, the imprecision of BMD as a measure of fracture risk [13] and the short-term follow-up period of supplementation trials are among the potential limiting factors when determining the effects of vitamin D on skeletal health.

An incomplete understanding of the mechanistic actions of how vitamin D, in particular 25(OH)D, mediates positive effects on the skeleton also contributes to the uncertainty for the need to recommend levels of serum 25(OH)D, level above which is required for renal 1,25(OH)_2_D synthesis. The association between eGFR and circulating 1,25(OH)_2_D levels in this elderly cohort is consistent with the role renal function has in determining vitamin D status [14]. Interestingly, while serum 1,25(OH)_2_D levels are determined by eGFR, in addition to PTH and 25(OH)D levels, no bone parameters were determined by serum 1,25(OH)_2_D levels, suggesting that changes to 25(OH)D levels were more important to determine MWT and BS/BV.

The strong positive relationship between serum 25(OH)D levels and MWT suggests that osteoblastic bone formation was improved in the presence of higher serum 25(OH)D levels. Serum 25(OH)D levels above 50 nmol/L significantly increase MWT. While it is possible that serum 25(OH)D levels of 70 nmol/L and above also increase MWT group (*p* = 0.021), when we adjusted for multiple comparisons, the significance was (*p* = 0.2), perhaps due to smaller number of participants per group. BS/BV, an indicator of texture and strength of bone, was negatively associated with 25(OH)D levels suggesting that patients with adequate vitamin D status had plate-like structure consistent with higher bone strength. Significantly lower BS/BV and Tb.Pf values were reported in a recent clinical study examining the effect of bisphosphonate treatment on femoral head trabecular bone micro-architecture in non-treated fracture controls as compared to hip-fracture patients. Authors concluded that lower values for both the variables suggest preserved connectivity of trabecular bone in the bisphosphonate group. More importantly, Tb.Pf and BS/BV were strongly correlated with measures of bone strength [15]. A lower BS/BV may also signify decrease bone resorption [15]; hence, a lower BS/BV with higher vitamin D levels observed in our study is consistent with structural effects of vitamin D on reducing bone resorption.

We have previously proposed, using animal and cell culture studies, that the direct supply of 25(OH)D to bone is required for local 1,25(OH)_2_D synthesis [5,16,17] and contributes to the regulation of bone formation [18] and resorption [19,20] processes. Few studies to date have investigated the relationship between 25(OH)D status and local metabolism of vitamin D within human bone. Elderly women with subcapital femur fracture have been shown to exhibit five-times lower levels of bone-derived 1,25(OH)_2_D levels when compared with aged-matched women without fracture, despite comparable circulating levels of 1,25(OH)_2_D [21]. We have previously shown that using eleven human biopsies, the relationship of CYP27B1, CYP24A1 and VDR mRNA levels with genes, including vitamin D responsive genes involved in bone remodeling [22]. Furthermore, we have shown that human primary osteoblasts synthesize 1,25(OH)_2_D from 25(OH)D via CYP27B1 activity, which directly promotes mineralization [23]. While these studies are indicative of the requirement for 25(OH)D to promote bone health, the question of changes in human structural bone in relation to serum 25(OH)D is less clear. In the present study, we have demonstrated that adequate serum 25(OH)D levels along with bone mRNA levels, which are higher for CYP27B1 and lower for CYP24A1, is associated with reduced BS/BV, which is indicative of a stronger trabecular bone structure. Our findings are concur with a recent clinical study in a Danish cohort of post-menopausal women that showed daily supplementation with vitamin D3 (2800IU) resulted in significantly improved bone strength but did not affect the areal BMD [24].

## 5. Conclusions

In conclusion, we have demonstrated that higher serum 25(OH)D levels are associated with improved bone formation. The association of serum 25(OH)D levels with bone quality variables of MWT and BS/BV, rather than bone mass variables, suggests that determining the efficacy of vitamin D supplementation on bone health should take in consideration measures of bone quality beyond bone mineral density. Furthermore, higher 25(OH)D along with higher expression of bone CYP27B1 and lower expression of CYP24A1 is associated with stronger plate-like structure and supports the paradigm of bone supply of 1,25(OH)_2_D levels to improve bone health.

## Figures and Tables

**Table 1:** Biochemical profile of the participants.

	Median (IQ Range)	Range
Age, years	86.5 (80.3–90.3)	63.0–102.7
eGFR, mL/min/1.73 m^2^	70 (47–84)	11.0–90+
Ionized Ca, mmol/L	1.19 (1.15–1.23)	1.03–1.37
25(OH)D, nmol/L	52.7 (30.4–73.4)	10–225
1,25(OH)2D, pmol/L	93.8 (67.1–129.8)	18.4–229
PTH, pmol/L	4.9 (3.28–9.43)	1.1–32.7
Osteocalcin, nmol/L	2.41 (1.21–3.24)	0.33–21.8
BS-ALP, µg/L	13.3 (9.78–17.23)	3.45–36.30

PTH: parathyroid hormone.

**Table 2:** Determinants of serum 1,25(OH)_2_D- Multiple linear regression analyses.

Dependent Variable	Independent Variables	Standardized Coefficients (beta)	*p*-Value
**Serum 1,25(OH)_2_D**	25(OH)D	0.332	<0.001
	+PTH	0.272	0.006
	+eGFR	0.439	<0.001
	+Constant	+0.187	0.992
		Combined *R*^2^ = 0.235	<0.001

**Table 3:** Multiple linear regression analyses for biochemical determinants of Mean Wall Thickness.

Dependent Variable	Independent Variables	Standardized Coefficients (beta)	*p*-Value
MWT	25(OH)D	0.745	<0.001
	+1,25(OH)_2_D	+0.095	0.190 (NS)
	+5.4		
		Adjusted *R*^2^ = 0.602	*p* < 0.001

MWT: Mean wall thickness.

**Table 4:** Multiple linear regression analyses for serum 25(OH)D, CYP27B1 and CYP24A1mRNA levels as determinants of Mean Wall Thickness.

Dependent Variable	Independent Variables	Standardized Coefficients (beta)	*p*-Value
MWT	25(OH)D	0.743	<0.001
	+CYP27B1	+0.043	0.655 (NS)
	+CYP24A1	+0.064	0.504 (NS)
	+5.1		
		Adjusted *R*^2^ = 0.54	*p* < 0.001

**Table 5:** Multiple linear regression analyses for serum 25(OH)D, CYP27B1 and CYP24A1mRNA levels as determinants of Bone Surface/Bone Volume.

Dependent Variable	Independent Variables	Standardized Coefficients (beta)	*p*-Value
BS/BV	25(OH)D	−0.294	0.012
	+CYP27B1	−0.208	0.115
	+CYP24A1	+0.306	0.021
	+27.5		
		Adjusted *R*^2^ = 0.104	0.016

BS/BV: Bone surface/bone volume.

## Appendix A

**Table A1:** Comparison of Mean Wall Thickness across 25(OH)D intervals (Kruskal-Wallis test).

25(OH)D Interval nmol/L (Number of Patients)	Median 25(OH)D Levels (nmol/L)	Test Statistics	*p* Value (vs. >70 nmol/L)	Adjusted *p* Value ^a^ (vs. >70 nmol/L)
10–30 (21)	6.17	−50.31	<0.001	<0.001
30–50 (18)	7.16	−34.77	<0.001	<0.001
50–70 (20)	8.40	−17.17	0.021	0.12
>70 (29)	9.70			

^a^ Significance values have been adjusted by the Bonferroni correction for multiple tests.
